# Complementary therapy use by patients and parents of children with asthma and the implications for NHS care: a qualitative study

**DOI:** 10.1186/1472-6963-6-76

**Published:** 2006-06-15

**Authors:** Alison Shaw, Elizabeth A Thompson, Debbie Sharp

**Affiliations:** 1 Academic Unit of Primary Health Care, Department of Community Based Medicine, University of Bristol, Cotham House, Cotham Hill, Bristol, England, UK; 2 Bristol Homeopathic Hospital, United Bristol Healthcare Trust, Cotham Hill, Bristol, England, UK

## Abstract

**Background:**

Patients are increasingly using complementary therapies, often for chronic conditions. Asthma is the most common chronic condition in the UK. Previous research indicates that some asthma patients experience gaps in their NHS care. However, little attention has been given to how and why patients and parents of children with asthma use complementary therapies and the implications for NHS care.

**Methods:**

Qualitative study, comprising 50 semi-structured interviews with a purposeful sample of 22 adults and 28 children with asthma (plus a parent), recruited from a range of NHS and non-NHS settings in Bristol, England. Data analysis was thematic, drawing on the principles of constant comparison.

**Results:**

A range of complementary therapies were being used for asthma, most commonly Buteyko breathing and homeopathy. Most use took place outside of the NHS, comprising either self-treatment or consultation with private complementary therapists. Complementary therapies were usually used alongside not instead of conventional asthma treatment. A spectrum of complementary therapy users emerged, including "committed", "pragmatic" and "last resort" users. Motivating factors for complementary therapy use included concerns about conventional NHS care ("push factors") and attractive aspects of complementary therapies ("pull factors"). While participants were often uncertain whether therapies had directly helped their asthma, breathing techniques such as the Buteyko Method were most notably reported to enhance symptom control and enable reduction in medication. Across the range of therapies, the process of seeking and using complementary therapies seemed to help patients in two broad ways: it empowered them to take greater personal control over their condition rather than feel dependant on medication, and enabled exploration of a broader range of possible causes of their asthma than commonly discussed within NHS settings.

**Conclusion:**

Complementary therapy use reflects patients' and parents' underlying desire for greater self-care and need of opportunities to address some of their concerns regarding NHS asthma care. Self-management of chronic conditions is increasingly promoted within the NHS but with little attention to complementary therapy use as one strategy being used by patients and parents. With their desire for self-help, complementary therapy users are in many ways adopting the healthcare personas that current policies aim to encourage.

## Background

Asthma is the most common chronic disease in the UK, constituting a considerable burden to patients, their families and the National Health Service (NHS) [1]. Many asthma patients experience significant morbidity and lifestyle limitations, despite general improvements in treatment standards [2]. Previous research has revealed that asthma patients experience problems with the quality of their NHS care, including gaps in information provision, for example about oral steroids [3,4]. Qualitative studies have indicated that asthma patients' treatment expectations may differ from those of clinicians, patients often do not adhere to treatment regimes, use their own coping strategies to manage their condition and expect clinicians to acknowledge their personal disease experiences [5-7].

Use of complementary therapies is increasing in the UK and in other western countries, often for chronic conditions [8,9]. Most UK use takes place outside of the NHS in the private sector. Patient demand for complementary therapies has been recognised within UK health policy [10]. While patients are choosing complementary therapies, many clinicians remain unconvinced of their value and debate the merit of providing greater NHS access to complementary therapies [11-13].

UK estimates of the prevalence of complementary therapy use for asthma vary widely, from 6% to 70%, with variations probably due to methodological problems in the studies [14,15]. For example, a survey of patient members of the charity 'Asthma UK' reported much higher complementary therapy use than a general population survey, but the sample was likely to have been atypical [16]. Both surveys had low response rates, with no information on non-responders [15,16]. Despite the absence of a reliable prevalence estimate, a sizeable minority of asthma patients seem to be turning to complementary therapies.

The clinical evidence is inconclusive regarding the effectiveness of complementary therapies for asthma, with systematic reviews noting the paucity of high quality randomised controlled trials [17-21]. Given the apparent mismatch between patients' treatment choices and the clinical evidence, it is important to know more about why patients are looking outside of the NHS, as this may reveal unmet needs and priorities, and contribute to the enhancement of patients' NHS asthma care.

Qualitative studies of complementary therapy use indicate that patients use such therapies because of dissatisfaction with conventional medicine, perceived harmful effects of conventional treatments, desire for a more holistic approach and greater philosophical congruence with complementary therapies [22,23]. Limited attention has been given to asthma patients' interest in complementary therapies in the context of self-management [24]. A focus group study of mothers of children with asthma from different ethnic backgrounds reported calls for greater access to complementary treatments [25]. A small number of studies have examined the treatment preferences of asthma patients using specific complementary therapies. For example, one study comparing patients using homeopathy and conventional asthma treatment found that homeopathy users expressed stronger preferences for doctors to treat them as a whole person [26]. But there has been little detailed investigation of patients' and parents' views of a variety of complementary therapies for asthma and how far the general motivating factors for complementary therapy use identified above translate to asthma specifically. Furthermore, there has been little consideration of patients and parents' perceptions regarding the impact of complementary therapies on asthma and the implications of complementary therapy use for NHS asthma care. These are some of the issues we wish to explore in this study.

In addition to mapping the range of views of complementary therapies among patients and parents of children with asthma, both users and non-users of complementary therapies, the main aims of this study were threefold:

• To investigate why and how patients and parents of children with asthma use complementary therapies as part of their coping strategies.

• To explore their experiences of the impact of complementary therapies on their condition.

• To consider the implications of the findings for the overall delivery of high quality asthma care within the NHS.

## Methods

Qualitative methods were used and the study took place in Bristol (England) during 2004. The Central and South Bristol Research Ethics Committee approved the study.

### Participants

The overall sampling strategy was purposeful [27] aiming to recruit participants who could provide rich information to address the research objectives. Within this, sampling for variation was used to identify patients and parents of children with asthma from a range of socio-demographic backgrounds and healthcare settings (NHS and non-NHS), using a variety of complementary therapies. We also included non-users of complementary therapies in the early stages of the sampling, to explore broader questions about patients' attitudes to complementary therapies, but these do not form the main focus of this paper.

Sampling comprised various stages. First we recruited two contrasting general practices, one in an affluent suburb with potential local access to private complementary therapy clinics (practice A), and one in a deprived inner city area with access to a subsidised complementary therapy service via the practice (practice B). Along with a patient information sheet and covering letters signed by the GP and the research team, a brief screening questionnaire on complementary therapy use was sent to a random sample of 100 patients from each practice. Patients included were aged seven plus, with an asthma diagnosis, and a prescription for asthma medication in the past 12 months. The initial sample size was based on an estimate of the proportion likely to respond to a community survey, to give a reasonable pool of patients to purposefully sample a heterogenous sample for interviews. In practice A, 32 patients returned questionnaires (32%), with 28 agreeing to be contacted for an interview. In practice B, 22 returned questionnaires (22%), with 17 agreeing to be contacted. From these, a sub-sample of patients with a range of ages and gender, both non-users and users of various complementary therapies were selected.

Subsequently, a purposeful sample of asthma patients and parents from a respiratory clinic at a children's hospital, a NHS homeopathic hospital, and private complementary therapists were approached via a doctor, nurse or therapist. They were given the same covering letters and information sheet and asked to indicate whether they were willing to be contacted by the research team. Those known to be complementary therapy users were selected as this stage, as data saturation had been reached with non-users from the general practices.

### Data collection

Semi-structured interviews were conducted by AS in participants' homes. A flexible topic guide was used, allowing participants to introduce new issues, and incorporating new lines of questioning in response to emerging themes as data collection and early analysis progressed [28]. Broad topics covered in all interviews included patients' experiences of NHS asthma care, views and decision-making about complementary therapies, and for users their experiences of particular therapies. Interviews with children incorporated prompts and pictures on a laptop computer to aid engagement. Interviews with adults lasted between 25 minutes to an hour. Paired interviews with children and parents lasted 30 minutes to 1.5 hours, the first part focusing on the child and the second part on the parent. All were recorded using a mini-disc recorder and transcribed verbatim by a research secretary.

### Data analysis

Preliminary analysis commenced alongside early interviews and progressed iteratively. A thematic approach was used, guided by the principles of constant comparison [29]. "Open" coding of individual transcripts generated an initial coding framework, which was added to and refined, with coded material regrouped and recoded as new data was gathered. The codes were gradually built into broader categories and through comparison across transcripts higher-level themes were developed. Data within themes were scrutinised for disconfirming and confirming views across the range of participants [28]. AS led the analysis. ET and DS coded a sub-sample of transcripts, and all discussed and agreed the coding framework and themes on a repeated basis during the data collection and analysis period.

## Results

Fifty interviews were conducted. The numbers of patients from each setting and their characteristics (socio-demographic and complementary therapy use) are shown in table 1. Thirty-one patients were using complementary therapies for asthma, six were using therapies for other health problems not asthma and thirteen were non-users. The majority of users were female, either adult patients or mothers choosing complementary therapies for their children. They were from a variety of socioeconomic backgrounds – while private therapies were mostly used by those from more affluent backgrounds, NHS-provided homeopathy was used by participants from diverse backgrounds and a minority of less affluent participants were accessing subsidised therapies via their general practice.

A summary of reasons for non-use of complementary therapies emerging from interviews with non-users is provided in table 2. Broadly speaking, non-users fell into three broad groups: first, those who were strongly sceptical about complementary therapies for reasons such as a lack of belief that such therapies work and a strong belief in "scientific" medicine; second, those who trusted and primarily wanted to follow the recommendations of conventional doctors and would only try complementary therapies if advised by them; and third, those who were interested in and open to trying complementary therapies but had not yet done so, for reasons such as lack of information, because they perceived complementary therapies to be relevant for other conditions but not for asthma, or had simply "not got round to it". Some of these patients and parents noted that certain "trigger factors" could potentially prompt them to turn to complementary therapies for asthma, for example if their prescribed medication was no longer controlling their symptoms.

## Nature of complementary therapy use: committed, pragmatic and last resort users

Complementary therapies were used in a range of ways by patients and parents, varying in the degree to which they played a role in patients' self-management of their health. Broadly speaking, complementary therapy users fell into three categories. First, complementary therapies were the first port-of-call, and the NHS the last resort, for a minority of participants who held "alternative" philosophical beliefs about health that often differed from those underpinning conventional medicine ("committed users"). While these patients and parents ideally wanted alternatives to conventional asthma medication, and had a preference for non-pharmaceutical treatments, none had stopped taking their prescribed medication, recognising the potential dangers of doing so. Other participants had a more pragmatic approach towards complementary therapies, seeking various treatments and self-help strategies to improve asthma symptoms and related health issues (for example, allergies and sleep problems) alongside their prescribed medication, without a strong philosophical preference for complementary therapies ("pragmatic users"). Finally, a small group of participants were using complementary therapies somewhat in desperation, after trying conventional treatments and finding them to be ineffective at managing their or their child's condition. Primarily, these were patients for whom medication had escalated, often with limited benefit for controlling symptoms ("last resort users"). Table 4 provides a typology of complementary therapy users along with the types of non-users described previously, identifying a spectrum from "sceptical non-users" through to "committed users".

This paper will focus on findings from the thirty-one participants using complementary therapies for asthma (twelve adults and nineteen child-parent pairs), as these provide the richest data to address the main study objectives. The themes presented are supported by data reflecting the full range of expressed views (tables 5, 6, 7, 8). Most data is from older children, parents and adult patients, as younger children provided less data relevant to these themes.

### How complementary therapies are accessed and used

The first broad theme concerns the ways in which complementary therapies are accessed and used, which fall into two categories: routes into complementary therapies and the nature of complementary therapy use.

#### Routes into complementary therapies: sources of information and "referral" routes

The complementary therapies used for asthma are summarised in table 3. Most common were Buteyko breathing techniques and homeopathy. Sources of information on complementary therapies and "referral" routes were primarily via word-of-mouth (for example, friends and family), health shops, the media, books or the internet. While a small number of patients had been referred to the NHS homeopathic hospital by their GP or accessed subsidised therapies via their general practice, complementary therapies were primarily accessed through networks of informal personal contacts, outside of the NHS. This included consultations with private therapists, purchase of over-the-counter from chemist or health shops, or self-help strategies without products or therapist contact (for example, self-taught breathing techniques). A few adult patients and parents were complementary therapists themselves, receiving treatments via mutual exchange between therapists with no financial payment.

### Motivating factors for complementary therapy use

The second broad theme concerns reasons for complementary therapy use, which fall into two categories: "push factors" from conventional medicine and "pull factors" from complementary therapies.

#### "Push factors" from conventional medicine

"Push factors" from conventional medicine can be viewed as problematic aspects of NHS care that cause asthma patients or parents to look elsewhere for help (see table 5). These included the following: concerns about drugs as standard first line treatment on diagnosis of asthma along with a lack of health promotion advice from NHS health professionals; concerns about side-effects and long-term dependence on asthma medication; and concerns about ongoing escalation of medication with limited benefit for controlling symptoms. Concerns about steroid use (both inhaled and oral) were the most prominent, including perceptions of connections with anabolic steroids and concerns about side effects. For example, a menopausal woman was concerned about the impact of steroids on bone density and osteoporosis, and a mother was concerned about what she perceived as abnormal body hair growth on her child as a result of taking steroids over a period of time. Thus, a range of concerns about long-term use of prescribed medication were the primary source of patients' and parents' discontent with conventional NHS care.

#### "Pull factors" from complementary therapies

The second group of reasons for complementary therapy use are attractive "pull factors" from complementary therapies (see table 6). These included the following: a desire for "natural" or non-invasive treatments; qualities of complementary therapy consultations (for example, a "holistic" approach); personal commitment to "alternative" philosophies of health; and prior experiences of benefit from complementary therapies for other health problems or asthma (a factor in initial and continued use for asthma respectively). Thus attraction to complementary therapies was derived both from factors "internal" to the patient or parent (personal philosophical beliefs) and to various "external" factors (perceived qualities of complementary therapies and therapists).

### The impact of using complementary therapies

The third broad theme concerns experiences of the impact of complementary therapies, or the opportunities that complementary therapy use provided, both for asthma specifically and for the person more broadly. Regarding impact on asthma symptoms, the therapy most commonly experienced as beneficial was breathing techniques, whether taught formally (for example, Buteyko) or self-taught. The main valued benefit was enhanced control over breathing without recourse to medication, along with reduced medication use (both preventative and "relievers"). While complementary therapies were not always experienced as effective for asthma symptoms, participants usually reported broader personal benefits from trying other ways of managing their condition. These fall into two categories: the benefits of self-help and taking control, and the exploration of a broader range of causes of asthma.

#### The benefits of self-help: taking control versus dependence

A key beneficial opportunity that complementary therapy use provided was to empower patients to help themselves (see table 7). Participants valued techniques that enabled them or their children to have greater personal control over their asthma, rather than being controlled by it. This particularly emerged from patients using breathing techniques (for example,. Buteyko) but was also apparent for other self-help based therapies (for example, Bowen therapy). Self-help strategies were either learned through consultations with complementary therapists or self-taught, and mostly comprised breathing and relaxation techniques. Parents particularly welcomed techniques that could help to stop the escalation of panic and relax their child, to control asthma attacks. Acknowledgement of the psychological or emotional dimensions of asthma was evident within some patients' and parents' desire to feel more in control. Some participants described the broader value of self-help in terms of inner benefits, making one "feel better inside", rather than simply removing outward physical symptoms. They valued taking responsibility for their own health, rather than being seen as a "victim" of their condition.

The flipside of valuing greater personal control was a desire for reduced dependence on prescribed medication. This emerged strongly across the range of participants. Patients and parents were not primarily using complementary therapies because asthma medication was experienced as ineffective, but because they disliked reliance on medication, especially steroids (both inhaled and oral). They wanted more advice on preventative self-help strategies to enhance their general health and reduce their "susceptibility" to asthma (for example, through diet, breathing techniques or homeopathy), along with less routine reliance on drug-based asthma management within their NHS care.

In parallel with using non-drug strategies, participants often self-managed asthma medication, aiming to reduce doses or frequency of use when possible. Parents often did this because they wondered if maintaining the prescribed dose masked underlying improvements in their child's condition. However, a tension between reducing medication and awareness of the potentially life-threatening nature of asthma was expressed and was experienced as a difficult pathway to navigate.

#### Exploring a broader range of causes of asthma

The second benefit of complementary therapy use was the provision of opportunities to explore a wider range of causes of asthma than usually discussed within their NHS care (see table 8). Patients and parents often desired more understanding of what was causing the asthma symptoms, including a broader range of potential triggers (for example, environmental, dietary and emotional). Some parents had wanted more information from their child's GP about asthma and its triggers at the time of diagnosis, using the internet to seek such information. They also sought answers to bigger questions relating to the apparent increase of asthma among children, including the role of environmental factors and the perceived "over-diagnosis" of asthma in primary care.

Using complementary therapies was one way that participants tried to gain deeper insight into a wider range of possible causes of asthma. Complementary therapy consultations, particularly homeopathy, were often valued for their broader perspective on causes and their attention to patients' underlying susceptibility to asthma, through "constitutional" treatments that went beyond surface symptoms. Patients and parents appreciated the opportunities that complementary therapy consultations provided to explore "sideline" issues such as sleeping and more general anxieties, which were perceived as potentially contributing to asthma but were not usually discussed within their NHS asthma consultations.

Training courses on the Buteyko method was experienced as helpful not only for teaching breathing techniques but for allowing discussion about what asthma is and various possible triggers, opportunities that were seen as often lacking within NHS settings. Patients and parents were rarely highly critical of their conventional care, recognising the constraints on NHS staff (for example, time). However, complementary therapy consultations were valued not only for giving longer time, but for the quality of that time, and for the acknowledgement that health problems such as asthma do not always have clear-cut, "scientific" answers.

Wider philosophical concerns about a "sticking plaster" approach within conventional medicine were also sometimes expressed. NHS care was perceived to be generally good at addressing patients' presenting symptoms, but poor at addressing deeper underlying causes of illness (for example, social and emotional factors). Through more holistic treatments, some patients and parents were exploring broader questions about their or their children's health that conventional medicine seemed unable to answer.

## Discussion

Complementary therapy use for chronic conditions is increasing in the UK. This study offers a qualitative account of why and how patients and parents of children with asthma are using complementary therapies and the perceived impact of complementary therapies on their condition. It highlights how that, with the exception of small pockets of NHS provision, patients and parents of children with asthma are primarily using complementary therapies outside of the NHS, identifying and accessing services via informal networks of personal contacts. It suggests that patients are using complementary therapies alongside not instead of conventional asthma treatment, with a spectrum of types of complementary therapy users, including "committed", "pragmatic" and "last resort" users.

Previous studies have identified problems that patients perceive with their asthma care, for example gaps in information about medication [3,4]. Our findings highlight some specific areas of concern that could be addressed within NHS consultations: concerns about long-term dependence on medication, concerns about side-effects of steroids (inhaled and oral), and the desire for advice on non-drug preventative strategies (for example, diet). Our findings go further to illustrate some potential consequences of these perceived problems. They demonstrate that certain concerns about conventional asthma care play a role in patients' and parents' decisions to look outside of the NHS, along with attractive aspects of complementary therapies. This supports the general categorisation of "push" and "pull" factors for complementary therapy use [22]. However, while previous studies cite broad dissatisfaction with conventional medicine as motivating complementary therapy use, this study indicates that asthma patients using complementary therapies are not so much dissatisfied with the overall quality of their conventional care, but have specific concerns about long-term use of prescribed medication, especially steroids (both inhaled and oral). Rather than rejecting conventional medicine wholesale, they are incorporating complementary therapy use and self-help techniques into their own coping strategies, to reduce their dependence on medication, be proactive in managing their or their child's condition and explore a broader range of causes of asthma.

Patients' and parents' desire for a more "holistic" approach to asthma supports the findings of previous research that asthma patients using a complementary therapy wish to be treated as "a whole person" [26] and adds weight to the argument that complementary therapies offer patients new ways of understanding their condition that go beyond "reductionist" explanations [30]. Previous research suggests that parents of children with asthma can feel "out of control" and develop strategies to take charge of their child's condition [31]. This study indicates that complementary therapy use is one such strategy. The desire for greater control expressed by participants in this study is not unique to complementary therapy users, other studies revealing asthma patients' wishes for more involvement in treatment decisions [32]. The need to increase patients' control over their condition has been recognised in asthma guidelines for several years [33]. However, this study indicates that if patients and parents do not experience such control through their conventional asthma care, they may look outside of the NHS to complementary therapies.

What are the implications of the findings for the overall quality of asthma care? Health policy makers and providers increasingly encourage patients with chronic conditions to take responsibility for their health and reduce their dependence on healthcare systems [1,34], illustrated by initiatives such as the 'Expert Patient' programme [35]. Through adopting proactive and creative self-management strategies, complementary therapy users are in many ways adopting the healthcare personas promoted by current policies. However, their strategies differ in at least one important way from those usually considered by healthcare providers. Guided plans for symptom monitoring and medication use have been seen as the main route to asthma self-management [36], although their value has been debated [37,38]. In contrast, patients who use complementary therapies are developing their own self-management techniques, prioritising non-drug approaches to control their condition. Future discussions about asthma self-management should take greater account of patients' own coping strategies and treatment priorities – whether conventional or complementary.

Current quality indicators for asthma management in primary care focus on various quantitative measures at a practice level, for example percentages of asthma patients who have had a review in the past fifteen months, or who smoke and who have received smoking cessation advicer [39]. While these outcomes are undoubtedly important from a clinical audit perspective, studies such as ours suggest that evaluations of asthma care may need to adopt broader definitions of quality, allowing more individualised patient-level qualitative outcomes that incorporate a broader range of their treatment preferences and needs.

Health professionals often express reservations about complementary therapies, including concerns about lack of evidence of effectiveness, safety and the quality of complementary therapists' practice [11,40]. Without questioning the legitimacy of some of these concerns, there may be aspects of patients' and parents' complementary therapy use that can be capitalised upon, in order to enhance the overall quality of asthma care. Openness to self-management and a willingness to take responsibility for one's health are examples of qualities that could be nurtured across a broader range of patients, beyond complementary therapy users, to the advantage of both NHS professionals and patients. The precise mechanisms for nurturing such qualities require further consideration. One possibility might be the development of more local 'expert patient' self-management programmes, tailored to patients and parents of children with asthma, contrasting with current generic programmes offering control mechanisms for patients with a variety of chronic conditions. However, the challenges of bringing about a cultural shift towards self-management, across a broad range of patients, are recognised.

The "push factors" for complementary therapy use identified in this study indicate several areas of NHS asthma care that could be improved. In particular, patients' and parents' concerns about long-term medication use reveal the need for greater discussion of the risks and benefits of conventional asthma treatment within NHS consultations. Furthermore, whatever an individual clinician's attitudes to complementary therapies might be, this study suggests that more open communication with patients and parents about complementary therapy use may be valuable. Initiating a conversation about complementary therapies could allow patients' and parents' concerns about conventional treatment, and the reasons why they are turning to complementary therapies, to be discussed, in order to improve their NHS care. This might enable concerns about both conventional and complementary treatments to be aired – both patients' concerns regarding conventional treatment (for example, regarding side-effects of asthma medication) and professionals' concerns about complementary therapies (for example, about potential interactions between complementary and conventional treatments).

A challenge for NHS practitioners is how to facilitate greater communication about complementary therapy use within asthma consultations, when patients may not disclose such self-care because of fears about health professionals' responses [41]. One simple way forward could be to build in structured prompts regarding patients' or parents' interest in or use of complementary therapies, for doctors and nurses conducting asthma consultations in primary care, both at diagnosis and at review. This may be particularly important for patients whose asthma is not well controlled with prescribed medication, whose medication has escalated over time, or are long-term users of preventer medication (inhaled or oral steroids), as our study suggests that these are the patients who may be particularly likely to look to complementary therapies for additional help.

Health professionals may feel uncomfortable initiating discussions about complementary therapies if they feel they lack relevant knowledge to advise patients, or if they perceive them to be ineffective or unsafe. However, patients exercise their autonomy and make decisions about healthcare (whether conventional or complementary) often independent of the evidence-based medicine principles that so govern NHS practice [42]. Thus, avoiding such discussion may only contribute to the continued situation of patients' undisclosed use of complementary therapies, which may potentially pose greater risks. At the least, providing patients and parents with opportunities to discuss the full range of their concerns, preferences and needs is likely enhance the overall quality of asthma care, particularly in an age of patient-centred medicine where ideals of choice and access to services are so valued.

Resources are beginning to be developed to aid clinicians in their search for information on the effectiveness of complementary therapies. An example is the CAMEOL database funded by the Department of Health, [43] which contains summaries of systematic review evidence on particular complementary therapies for specific conditions. Such resources are as yet in their infancy, and one recommendation of this study is that they include high quality information on therapies relevant for asthma as well as the other conditions prioritised to date (for example, cancer and mental health), given that asthma is the most common chronic condition in the UK, consuming considerable NHS resources [1].

In qualitative terms the findings of this study are likely to be transferable to asthma patients and parents using complementary therapies from similar backgrounds and settings in other parts of the UK. The majority of complementary therapy users were female, mirroring findings of previous research. Patients from ethnic minorities were under-represented. Other authors have begun to explore attitudes to complementary therapies among South Asians with asthma and mothers of children with asthma from various ethnic backgrounds [24,25]. Examination of cultural aspects of complementary therapy use within specific ethnic groups may be beneficial in the future. The findings of this study have fed into the design of a questionnaire that is being used in a survey of the prevalence of complementary therapy use among asthma patients in primary care, which will examine the generalisability of the qualitative findings regarding predictors of use.

## Conclusion

Complementary therapy use among patients with chronic conditions is increasing. Patients and parents of children with asthma are turning to complementary therapies because of various concerns about conventional NHS asthma care (primarily concerns about long-term medication use, especially steroids) and because of attractive aspects of complementary therapies. Breathing techniques such as the Buteyko Method are of particular interest to patients and parents, and are experienced by some as beneficial for enhancing breathing control and enabling reduction in asthma medication. The process of seeking and using complementary therapies appears to help patients in two broad ways – empowering them to take greater personal control over their condition and enabling exploration of a broader range of causes of asthma than are discussed within their NHS care.

While health professionals often express reservations about the clinical value of complementary therapies, complementary therapy use seems to be indicative of an underlying desire among patients and parents for greater self-care and need for opportunities to address their concerns regarding NHS asthma care. Self-management of chronic conditions is increasingly promoted within the NHS but to date there has been little recognition of the role that complementary therapies may play in patients' own self-management strategies. With their desire for greater self-help, complementary therapy users are in many ways adopting the healthcare personas that current policies aim to encourage. Openness to self-management and a desire to take responsibility for one's health are qualities that could fruitfully be nurtured across other patient groups beyond complementary therapy users, to the advantage of both NHS professionals and patients.

## Competing interests

EAT is a consultant homeopathic physician at one of the recruitment settings used for this study. The authors have no other competing interests.

## Authors' contributions

All authors contributed to the design, analysis and writing-up of this study. AS was responsible for day-to-day management and conduct of the study, conducted the interviews, led the analysis and produced the first draft of the manuscript. All authors have read and approved the final manuscript.

## Pre-publication history

The pre-publication history for this paper can be accessed here:



## Figures and Tables

**Table 1:** Recruitment settings and participants

Practice A: general practice in an affluent sub'urb with potential access to local private complementary therapy clinics:	
	23 patients
	11 adults, 12 children (with 12 mothers)
	12 female, 11 male patients
	7 using complementary therapies for asthma
Practice B: general practice in a deprived inner city area with access to subsidised complementary therapies via the practice:	
	10 patients
	6 adults, 4 children (with 3 mothers and 1 father)
	7 female, 3 male patients
	7 using complementary therapies for asthma

Outpatient respiratory clinic at a NHS children's hospital:	
	5 patients
	All children (with 4 mothers and 1 father)
	2 female, 3 male patients
	All using complementary therapies for asthma

Outpatient clinic at a NHS homeopathic hospital:	
	5 patients
	All children (with 5 mothers)
	2 female, 3 male patients
	All using complementary therapies for asthma

Private complementary therapists:	
	7 patients
	4 adults, 3 children (with 3 mothers)
	4 female, 3 male patients
	All using complementary therapies for asthma

**Table 2:** Reasons for non-use of complementary therapies

• Concern about lack of scientific evidence that complementary therapies work
• A strong belief in the value of "scientific medicine"
• Conventional medicine works for asthma: "if it ain't broke don't fix it"
• No belief in complementary therapies: "old wives' tales", "myth", "superstition"
• Strong trust in doctors: "the doctor knows best"
• Lack of awareness of or interest in complementary therapies
• No perceived need: "my asthma's not that severe"
• No complementary therapy use within personal social networks
• Financial cost of private complementary therapies
• Uncertainty about quality and safety of over-the-counter complementary treatments

**Table 3:** Complementary therapies used for asthma

Acupuncture	Kinesiology
Aromatherapy	Massage
Bowen Therapy	Nutritional therapy (and general changes to diet)
Breathing techniques (e.g. Buteyko Method)*	Osteopathy/cranial osteopathy
Chiropractic	Reflexology
Flower remedies	Relaxation
Homeopathy*	Visualisation/guided imagery
Herbal medicine	Yoga (including yoga breathing)

*Therapies most commonly used

**Table 4:** Typology of non-users and users of complementary therapies (CTs) for asthma

**Non-users**			**Complementary therapy (CT) users**		
***CT sceptics: ***do not believe that CTs work; strong belief in value of "scientific" medicine	***Trust the doctor: ***only try what the doctor recommends; would only use CTs if the doctor advises	***Open or interested non-users: ***open to using CTs but not yet tried; certain "trigger factors" could prompt use	***Last resort users: ***tried all conventional treatments first; escalation of medication with lack of benefit, so turn to CTs	***Pragmatic users: ***"shop around" to use whatever treatments will help in parallel, both CTs and conventional medicine	***Committed users: ***CTs are preferred first port-of-call; committed to "alternative" philosophies of health

**Table 5:** 'Push factors' from conventional medicine

*Concerns about drugs as first line treatment on diagnosis of asthma with lack of health promotion advice*

"First line treatment was, 'We'll give you this medication and come back in a month's time and see how you feel and see if it's working'..but not very much of any other advice, no health promotion issues at all..I would expect 'These are preventative things you can do, and perhaps these may help you'." *(AA40, adult female, using yoga)*
*Dislike long-term dependence on medication, particularly steroids*

"I'm dependent on medicine, and it is a little bit scary..I didn't really want to be dependent on it, so I've tried to come off that and I've tried other things..I've cut out milk and wheat, and I've been to see a homeopathic doctor." *(CA02, adult female, using nutritional therapy and homeopathy)*

AS: So what do you feel about taking the medication? HY01: I don't think it's a good idea and it's really degrading like taking pills all the time. AS: In what way do you mean? HY01: It like makes you feel ill, it's a reminder sitting there saying you're ill and it's not nice, it puts you down taking pills." *(girl aged 15, using homeopathy and other therapies)*

TA06: "I'm not sure about these steroids, you know, I went on the steroids, lost my voice, so then I encountered this thing called Yamoa, I went to the local health place and took Yamoa and that helped..I wasn't really sure about taking the steroids, actually I didn't want to. AS: What was it that you felt uncertain about? TA06: Though they told me that it wouldn't affect my bones, I wasn't sure how right that really was, and I'm at the stage of life where osteoporosis is a thing..so I wasn't sure, I just don't like the idea of taking steroids." *(adult female, using herbal medicine)*

*Concerns about escalation of medication, with limited benefit for controlling symptoms*

"Over the years I've kept going to the doctor's and the asthma nurse and my medication has increased so much, but I never seem to get any better for it. If I had a course of steroids because I was really bad, then obviously that would boost me up, but I never got to the stage where I needed less medication, it was always more and more and more, let's try this inhaler, let's try these tablets." *(TA01, adult female, using Buteyko)*

"he was a poorly child and his asthma was starting to be very limiting, and it was the fact that it was getting worse, and you suddenly stop and think, this is all, you know, he's getting worse, inhalers don't really do anything..and he would need quite a lot of nebulising, and we had quite a lot of crises..and so we went to this homeopath." *(AY16, mother of boy aged 14, using cranial osteopathy and homeopathy)*

**Table 6:** 'Pull factors' from complementary therapies

*Desire for "natural" or "non-invasive" treatments*

"I prefer homeopathic mainly because it's not chemical and it's more natural to me, which I think is important from the point of view of the body as a whole. I think we should use nature" *(HY01, mother of girl aged 15, using homeopathy and other therapies)*

"What led me to it (reflexology) was the fact that it was non-invasive, you know, you weren't taking any more drugs" *(TA04, adult female, using various therapies)*
*Qualities of complementary therapy consultations: holistic approach, listening and time*

"it's time in the sense that they have got longer, but also they appear to be more interested. I like our GPs enormously and they're very talented individuals, but they don't have the time to talk..(homeopaths) look at the whole thing and they will say about diet, they will say what about your bedding, what about this, have you changed that?" *(AY29, mother of boy aged 13, using homeopathy)*

*Personal commitment to "alternative" philosophies of health*

"I'll carry on with complementary medicines and only use western as like a last resort, because...complementary medicine's putting things back where they should be, whereas western medicine's just tampering and putting things there that shouldn't be." *(HY01, girl aged 15, using homeopathy and other therapies)*

"the energy system is either sort of surrounding or intrinsic to the body in some way..it is like a blue print for the physical body..When our plans are ok we've got this great body, but our energy system, our plans keep getting attacked, they're being attacked by stresses, by toxins that we consume..by emotional stresses..and these plans are forever being distorted..so the physical body can't read the plans anymore. So physical dysfunctions, ailments are, I see it, as a result of distortions in the energy field." *(TC05, mother of girl aged 8, using kinesiology and homeopathy)*

*Experiences of effectiveness of complementary therapies*

"From the second night (of the Buteyko course) I stopped taking Ventolin because I controlled it with these exercises, and that's the first time I've been reliever-free in, well in 30-odd years. So it had quite a dramatic effect..I did the course at the end of last year..then in March I stopped the steroid as well." *(TA02, adult male, using Buteyko)*

AY16: "The last few years it's been a lot better. AS: Any reasons for that, do you think? AY16: I had homeopathy and (to mum) what's that other thing? Mum: Craniosacral therapy. AY16: Yes, that's it, and I think it helped a lot. AS: In what ways did you notice it helped? AY16: Just things like, I could run around without needing my inhaler, better at nights, not waking up as much, just generally I didn't need my inhaler as much and I could do a lot more" *(boy aged 14, using craniosacral osteopathy and homeopathy)*

**Table 7:** The impact of using complementary therapies: benefits of self-help and taking control

"I just started to think 'How can I get a grip on this?' and I worked out that it was for me, as helpful and perhaps sometimes more helpful, to kind of control when my asthma kicks in, to control my breathing rather than reaching for Ventolin, and ever since then I've not really needed to take any medicine, it's sort of breathing controlled..it's just put me in control of it." *(AA31, adult female, using self-taught breathing techniques)*

I wanted more control of my asthma because my drugs were doing that really..just to have better control without drugs." *(TA02, adult male, using Buteyko)*

"I'd heard about (Buteyko), and I've read about it, I just felt it would be nice to have something that he could do that would sort of empower him in a way, which it did seem to do" *(DC05, mother of boy aged 7, using Buteyko and homeopathy)*

"I just stumbled across this Buteyko method of breathing..about control, taking control of it rather than letting it take control of you, and learning how to work through it rather than progressively getting more panicky and out of breath and perpetuating the thing..I certainly feel that if she (daughter) starts getting an attack that she knows how to control it....I think there's a psychological element to it as well, where if you can take control at least part of it yourself, and you feel more in control, you can sort of cope with it more...the controlling of things yourself has got to be a good thing." *(DY03, father of 13 year old girl, using Buteyko)*

"I think self-help's better than being drugged..it's a lot better if you help yourself because it makes you feel properly better, if that makes sense, like it makes you feel better inside, as well as having the symptoms gone away, because you know you've taken them away yourself." *(HY01, girl aged 15, using homeopathy and other therapies)*

**Table 8:** The impact of using complementary therapies: exploring a broader range of causes of asthma

"GPs, because they're more science-based..I feel they're always looking for a scientific answer and there isn't always a scientific answer. Sometimes I think you need to look a bit broader..the more questions you ask the broader outlook you can have on something." *(HY01, parent of girl aged 15, using homeopathy and other therapies)*

"I went to the homeopath and..they deal with your susceptibility, your anxieties, the way that you feel with school, the way that you're sleeping..all the little sideline things that will help the susceptibility to having an asthma attack" *(DC05, mother of boy aged 7, using homeopathy and Buteyko)*

"She (Buteyko teacher) gave us a bit of background into asthma, and that was really helpful because I've never had a chance to sit down with a doctor, for him to tell me what it actually was or even an asthma nurse..she spoke about triggers and how different triggers, different foods, different atmospheres, trigger different people, so the background information that she gave was really helpful." *(TA02, adult male, using Buteyko)*

"I do believe that conventional medicine really, the NHS is just sort of..this sticking plaster approach of trying to keep people, placate people with drugs really..they're not saying, 'Well hang on a minute, why has this happened..why have we got so much asthma?" *(TC05, mother of girl aged 8, using kinesiology and homeopathy)*

there's an awful lot of things which conventional medicine is good at, but there's an awful lot of things that it isn't really very good for, because they are only treating the result and not the cause, so if you are only dealing with the symptoms and never the cause, you are going nowhere." *(AY16, mother of boy aged 14, using cranial osteopathy and homeopathy)*
